# Recrudescence de la fièvre jaune au Tchad: à propos du dernier cas confirmé dans le district sanitaire de Lai-Tchad

**DOI:** 10.11604/pamj.2021.38.248.27123

**Published:** 2021-03-08

**Authors:** Oumaima Mahamat Djarma, Djoissenanbaye Elisee, Mahamat Ali Bolti, Djiddi Ali Sougoudi, A Blondin Diop, Fatime Ahmat Haggar, Abdelsadick Hidjab, Adawaye Chatté, Joseph Mad-Toingue, Henri Fissou, Mekonyo Kolmian, Ali Mahamat Moussa

**Affiliations:** 1 Ministère de la Santé Publique et de la Solidarité Nationale, N´Djamena, Tchad,; 2 Hôpital Provincial de Farcha, N´Djamena, Tchad,; 3 Délégation Sanitaire de Lai-Tchad, Lai, Tchad,; 4 CHU de la Renaissance, N´Djamena, Tchad,; 5 Maison Médical de Dakar, Dakar, Sénégal,; 6 CHU de la Référence, N´Djamena, Tchad,; 7 Laboratoire Mobile de N´Djamena, N´Djamena, Tchad

**Keywords:** Fièvre jaune, recrudescence, Tchad, à propos d’un cas, Yellow fever, recrudescence, Chad, case report

## Abstract

La fièvre jaune (FJ) est une fièvre hémorragique virale, causée par le virus de la fièvre jaune, transmise par les moustiques du genre Aedes. Au Tchad depuis 2013, 04 cas de fièvre jaune ont été identifiés et confirmés dans le cadre du programme national de surveillance de la fièvre. Nous rapportons ici l'observation clinique du dernier cas confirmé dans le district sanitaire de Lai. Il s'agit d'un homme de 57 ans sans antécédents médicaux et chirurgicaux significatifs et un statut vaccinal inconnu. Il a consulté le 21 avril 2020 pour fièvre, ictère d'épistaxis d'abondance modérée à faible et hépatomégalie douloureuse. Dont les explorations para-cliniques ont permis de mettre en évidence le virus de la fièvre jaune en post mortem par RT-PCR. Ainsi la confirmation de la fièvre jaune dans ce district, le faible niveau de couverture vaccinale, la réalité de la circulation du virus et la présence du vecteur dans le pays devraient avertir d'une réelle menace de ré-émergence de la fièvre jaune au Tchad.

## Abstract (en)

Yellow fever (YF) is a viral haemorrhagic fever caused by yellow fever virus transmitted by Aedes mosquitoes. Since 2013, in Chad, four cases of yellow fever have been detected and confirmed as part of the national fever surveillance program. We here report the last clinical case confirmed in the health district of Lai. The patient was a 57-year-old man with no significant medical and surgical history and unknown immunisation status. He consulted on April 21^st^, 2020 for fever, moderate to low abundance jaundice and epistaxis (nosebleed) and painful hepatomegaly. Paraclinical examinations, such as RT-PCR, objectified yellow fever virus in post-mortem tissue sample. Thus, confirmed yellow fever cases in this district, the low level of vaccination coverage, the circulation of the virus and the presence of vector in the country should warn of a real threat of reemergence of yellow fever in Chad.

## Introduction

La fièvre jaune (FJ) est une fièvre hémorragique virale, due au virus amaril, transmise par des moustiques du genre *Aedes* [1]. Elle se présente sous des formes variées, de la simple fièvre, à l'ictère associé à des hémorragies diffuses avec une mortalité des cas graves allant de 20 à 50% [2]. Plus de 900 millions de personnes sont concernées dans 45 pays, dont 32 en Afrique et 13 en Amérique. On estime chaque année à 200 000 le nombre de cas et à 30 000 le nombre de décès dus à la fièvre jaune [3]. En Afrique, la FJ est endémique en zones de forêt en raison d´un cycle enzootique sauvage [4]. Au Tchad, en 2013 deux cas de fièvre jaune ont été identifiés et confirmés dans le cadre du programme national de surveillance de la fièvre jaune lors d´une flambée de la maladie dans la région frontalière du Darfour (Soudan). Par ailleurs, 139 cas suspectés et 9 décès ont été signalés grâce à cette surveillance renforcée [5]. Depuis lors le pays a enregistré 4 cas confirmés dont un cette année. Nous vous rapportons ici l´observation clinique du dernier cas confirmé dans le district sanitaire de Lai.

## Patient et observation

Il s´agit de Mr K.M, âgé de 57 ans, il consulte le 21 avril 2020 fièvre, jaunisse épistaxis de moyenne à faible abondance survenue les 14 et 17 avril 2020 et une hépatomégalie douloureuse. Il est sans antécédent médico-chirurgical notable et un statut vaccinal inconnu. Dans le mois avant le début de sa maladie, il n´aurait selon la famille effectué aucun déplacement en dehors de la ville de Lai. Son dernier voyage en date remonte à novembre 2019.

L´examen clinique réalisé lors de son hospitalisation à l´hôpital de Bebaloum a mis en évidence un ictère conjonctival, une hépatomégalie douloureuse flèche hépatique à 22 cm. Devant ces signes, une notification d´ictère fébrile est faite au système et le prélèvement fait ce jour même qui a mis en évidence en post mortem par RT-PCR le virus amaril; la recherche des trophozoïtes, des hépatites notamment le B and C sont aussi négatif. La recherche des autres arboviroses n´était pas réalisée. La numération a mis en évidence une hyperleucocytose 27.103/mm^3^ à prédominance polynucléaire neutrophile 18.103/mm^3^. Les hémocultures n´ont pas été réalisées.

Durant son hospitalisation il avait bénéficié d´une prise en charge médicale à base d´amoxicilline 3g/24h, Métronidazole 1,5g/24h et une Réhydratation 3l/24h. La recherche active des cas d´ictère fébrile dans la communauté a été faite au cours de l´enquête de couverture vaccinale et a consisté à rechercher dans les ménages visités les sujets présentant une fièvre avec un ictère et/ou accompagnement de saignement. Elle a permis de détecter et prélever cinq cas suspects. L´évolution clinique était marquée par la persistance des saignements et une trouble de la conscience score Q SOFA à 3. C´est dans ce contexte que le décès survient après 5 jours d´hospitalisation.

## Discussion

Dans notre cas clinique, la mise en évidence du virus amaril constitue une première dans ce district. Cependant, nous avons eu des limites notamment la recherche des autres arboviroses, et l´investigation entomologique. Situé au cœur de la province de la Tandjilé dans le sud du pays, le district de Lai occupe la partie centrale. Le réseau des pistes est relativement dense mais peu praticables en saison pluvieuse. Ceci rend une bonne partie inaccessible pendant au moins 5 mois chaque année. La population est estimée en 2020 à 20,428 habitants [6]. Ce cas mortel de fièvre jaune était le premier cas notifié dans la province où la couverture vaccinale antiamarile dans la population générale est inférieure à la cible fixée de 80% [7, 8].

En outre, elles sont en constante baisse aussi bien à l´échelle du district que dans les deux centres de santé de la ville de Lai. Cette situation ne permet pas d´assurer une immunité suffisante capable d´interrompre la transmission de la fièvre jaune au sein de la communauté. Aussi convient-il de signaler que la vaccination de routine est faite en stratégie porte à porte. Cette stratégie n´est pas adaptée à la vaccination de routine et n´offre pas de chance de vacciner des enfants avec les vaccins lyophilisés comme le vaccin antiamarile. La FJ est endémique dans certaines régions du monde 34 pays d´Afrique (Tanzanie, Zambie, Cote d´Ivoire, Sénégal, Cameroun, Gabon ...) et 13 en Amérique latine [8]. C´est une pathologie ré-émergente [9].

Des enquêtes entomologiques réalise dans certaines provinces du pays ont permis de mette en évidence la présence du vecteur *Aedes* [10]. Chez notre patient, tout porte à croire qu´il a eu un cycle de transmission urbain de la FJ. Ce cycle implique une transmission par des moustiques diurnes qui piquent préférentiellement l'homme (moustiques « domestiques »), comme *Aedes aegypti* en Afrique, ainsi que les *Hæmagogus* et *Sabethes* en Amérique du Sud. *Aedes aegypti* se développe dans les récipients et tout objet pouvant retenir de l'eau stagnante.

La fièvre jaune dépend alors de plusieurs variables: la densité humaine (urbanisation croissante), les déplacements humains (personnes non immunisées, moyens de transport emportant des moustiques), les zones péri-urbaines dégradées (favorisant le lien entre zones sylvatique et urbaine), le relâchement des précautions d'hygiène et de vaccination, le réchauffement climatique et les pluies excessives. Il s'agit d'autant de facteurs qui peuvent augmenter la transmission de la fièvre jaune [4].

## Conclusion

Ce cas confirmé de fièvre jaune n´est pas un cas isolé au Tchad. D´autre cas ont été notifié depuis 2013. Ainsi la confirmation de la fièvre jaune dans ce district, le faible niveau de la couverture vaccinale, la réalité de la circulation du virus et la présence du vecteur dans le pays devraient alerter d´une réelle menace de ré-émergence de la fièvre jaune au Tchad. Les recommandations immédiates ont été axées sur une vaccination de masse des populations de la province et un renforcement du système de surveillance épidémiologique active sur tout le territoire.

## Remerciements

A l´équipe de l´OMS, Dr Sorgho E et Dabi Esther pour le soutien technique. Aux points focaux: Nededjimbaye Marcel Blam Sitikreo Garandji Ndoubahidi Dorom Balamsia Sountou Keuziackae Ladibo pour leur collaboration.
